# Bending-loss-independent operation of slope-assisted Brillouin optical correlation-domain reflectometry

**DOI:** 10.1038/s41598-018-26153-6

**Published:** 2018-05-18

**Authors:** Heeyoung Lee, Tianyi Ma, Yosuke Mizuno, Kentaro Nakamura

**Affiliations:** Institute of Innovative Research, Tokyo Institute of Technology, 4259 Nagatsuta-cho, Midori-ku, Yokohama, 226-8503 Japan

## Abstract

We demonstrate loss-insensitive operation of slope-assisted Brillouin optical correlation-domain reflectometry by employing a special silica fiber with low bending loss. As fundamental characterization, we measure the coefficients of the power-change dependencies on strain and temperature to be 1.42 × 10^−4^ dB/µε and 3.28 × 10^−3^ dB/K, respectively. Subsequently, by comparing the distributed strain and temperature measurement results using a standard silica fiber and the special fiber, we show that this configuration offers highly stable loss-insensitive operation for practical use in the future.

## Introduction

Distributed strain and temperature sensing based on Brillouin scattering^1^ in optical fibers has been one of the major targets of research in the community of smart materials and structures. Various configurations have been reported thus far, including Brillouin optical time-domain analysis (BOTDA)^2–7^, Brillouin optical frequency-domain analysis (BOFDA)^8,9^, Brillouin optical correlation-domain analysis (BOCDA)^10–15^, Brillouin optical time-domain reflectometry (BOTDR)^16–20^, Brillouin optical frequency-domain reflectometry (BOFDR)^21^, and Brillouin optical correlation-domain reflectometry (BOCDR)^22–30^. Each configuration has different merits and demerits; for instance, the time-domain techniques generally have long measurement ranges, but their spatial resolutions are limited to sub-meter order^31^, though some recent papers have reported significantly enhanced resolutions^32,33^. In an analogous fashion, the correlation-domain techniques have random accessibility^12^ and extremely high spatial resolutions of up to millimeter-order^14^, but they suffer from limited measurement ranges^23^, although some enhancements have been reported^24^. Here, let us focus on BOCDR.

Since the basic idea was first implemented^22^, BOCDR has been extensively studied for more than ten years, leading to substantial improvement of its performance^24–30^. Some of the recent advances include the development of simplified configurations^25^, polymer-fiber-based configurations^27^, and high-speed configurations, such as phase-detected BOCDR^28,29^ and slope-assisted (SA-) BOCDR^30^. In SA-BOCDR, the Brillouin frequency shift (BFS), which corresponds to the information on the applied strain and temperature, is converted into the spectral power on the slope of the Brillouin gain spectrum (BGS). In addition to the high-speed operation^30^, SA-BOCDR has some unique features, such as the detection capability of the shortest-ever strain (<2 mm) below the nominal spatial resolution^34,35^ and the sensitivity on not only strain and temperature but also locally applied loss^30^. The latter feature is sometimes beneficial^36^, but from the viewpoint of stable measurement of strain and temperature, the final output of SA-BOCDR should not be influenced by unintended loss applied to the sensing fiber.

In this work, we demonstrate loss-insensitive operation of SA-BOCDR by employing a special silica fiber with low bending loss. We characterize the sensing performance of the loss-insensitive SA-BOCDR and find that the coefficients of the power-change dependencies on strain and temperature are 1.42 × 10^−4^ dB/µε and 3.28 × 10^−3^ dB/K, respectively (when the spatial resolution is 144 mm and the measurement range is 14.7 m). Subsequently, by comparing the distributed strain and temperature measurement result using the special fiber with that using a standard silica single-mode fiber (SMF), we prove the effectiveness of this loss-insensitive configuration.

## Results

### Principle

Brillouin-based distributed sensors including BOCDR basically utilize the BFS dependencies on strain and temperature. The strain- and temperature-dependence coefficients in silica SMFs at 1550 nm are reported to be approximately 500 MHz/% and 1.1 MHz/K, respectively^37,38^. To perform a distributed measurement, BOCDR exploits what we call a correlation peak, at which the Brillouin-scattered light is selectively observed. The correlation peak is generated by sinusoidal modulation of the laser output frequency, and its position can be arbitrarily set along the fiber under test (FUT) of the system^10,22^. If the modulation frequency *f*_m_ is swept, the correlation peak is scanned along the FUT, which provides distributedly measured BFSs. According to the theory^23^, the measurement range of the basic scheme is limited to

1 $${{d}}_{{\rm{m}}}=\frac{{c}}{2{n}{{f}}_{{m}}}$$

where *c* is the velocity of light in vacuum and *n* is the refractive index of the fiber core; the spatial resolution is given by

2 $${\rm{\Delta }}{z}=\frac{{c}\Delta {{v}}_{{B}}}{2{\pi }n{{f}}_{{m}}\Delta {f}}$$

where Δ*v*_B_ is the Brillouin bandwidth (= ~30 MHz in silica SMFs) and Δ*f* is the modulation amplitude.

In standard BOCDR schemes, to obtain the strain or temperature information at one sensing position along the FUT, the whole BGS needs to be acquired and then the BFS, at which the spectral power becomes the highest, is derived, resulting in a relatively time-consuming process. To solve this problem, in SA-BOCDR^30^, instead of the BFS itself, we use the change in the spectral power on the BGS slope (at a constant frequency *v*_B0_), which is in one-to-one correspondence to the BFS. This configuration is free from the time-consuming frequency-sweeping process, leading to much higher-speed operation. The optimal value of *v*_B0_, which is determined by differentiating the BGS^30^, is 10.85 GHz for standard silica SMFs. The coefficients of the power-change dependencies on strain and temperature in standard silica SMFs are reported to be 1.95 × 10^−4^ dB/µε and 4.42 × 10^−3^ dB/K, respectively^30^ (when the spatial resolution is 88 mm and the measurement range is 12.9 m), which are in moderate agreement with the values theoretically calculated from the BFS dependencies and the BGS shape^30^.

### Experimental setup

**Figure 1:**
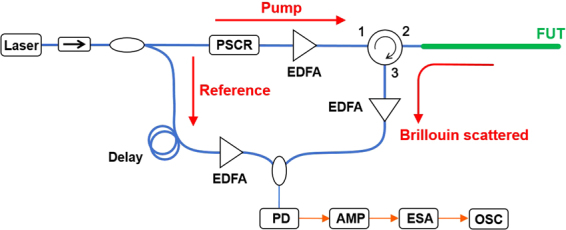
Schematic of the SA-BOCDR setup. AMP: electrical amplifier, EDFA: erbium-doped fiber amplifier, ESA: electrical spectrum analyzer, FUT: fiber under test, OSC: oscilloscope, PD: photodiode, PSCR: polarization scrambler.

Figure 1 shows the experimental setup of SA-BOCDR, which is basically the same as that previously reported^30^. The pump light was amplified to ~26 dBm using an erbium-doped fiber amplifier and injected into the FUT. The reference light was passed through a ~1-km-long delay fiber, amplified to ~2 dBm, and coupled with the Brillouin-scattered light (amplified to ~1 dBm) for heterodyne detection. By inserting a polarization scrambler in the pump path, the polarization-dependent signal fluctuations were suppressed to improve the signal-to-noise ratio (SNR). The heterodyned optical signal was converted into an electrical signal with a photodiode, amplified by 23 dB using an electrical amplifier, and observed with an electrical spectrum analyzer (ESA) (video bandwidth: 3 kHz, resolution bandwidth: 10 MHz) as a BGS. Exploiting the narrow band-pass filtering function of the ESA, the power change at a fixed frequency (=10.81 GHz; calculated by analyzing the measured BGS, as mentioned below) on the BGS slope was sequentially output to an oscilloscope, on which averaging was performed 1024 times. The modulation amplitude Δ*f* was set to 1.3 GHz. For distributed measurements, the modulation frequency *f*_m_ was swept from 6.91 to 7.10 MHz (note that, in a single-point measurement for characterizing the system performance, *f*_m_ was fixed at 7.04 MHz, which corresponds to the location of the correlation peak 7.9 m away from the circulator). According to Eqs (1and 2), these conditions resulted in the nominal spatial resolution of 144 mm and the measurement range of 14.7 m. The room temperature was 21 °C.

The low-bending-loss silica fiber used in the experiment was a 12.0-m-long trench-index-type special single-mode fiber (SMF; FutureGuide-BIS-B, Fujikura)^39,40^ with a low bending loss (e.g., 0.018 dB/turn at 1550 nm for a bending radius of 7.5 mm). One end of this special fiber was connected to the 1.0-m-long pigtail of the second port of an optical circulator via an “FC/APC” adaptor, and the other end was cut with an angle and immersed into index matching oil to suppress the Fresnel-reflection-dependent signal fluctuations.

### Fundamental characterization

**Figure 2:**
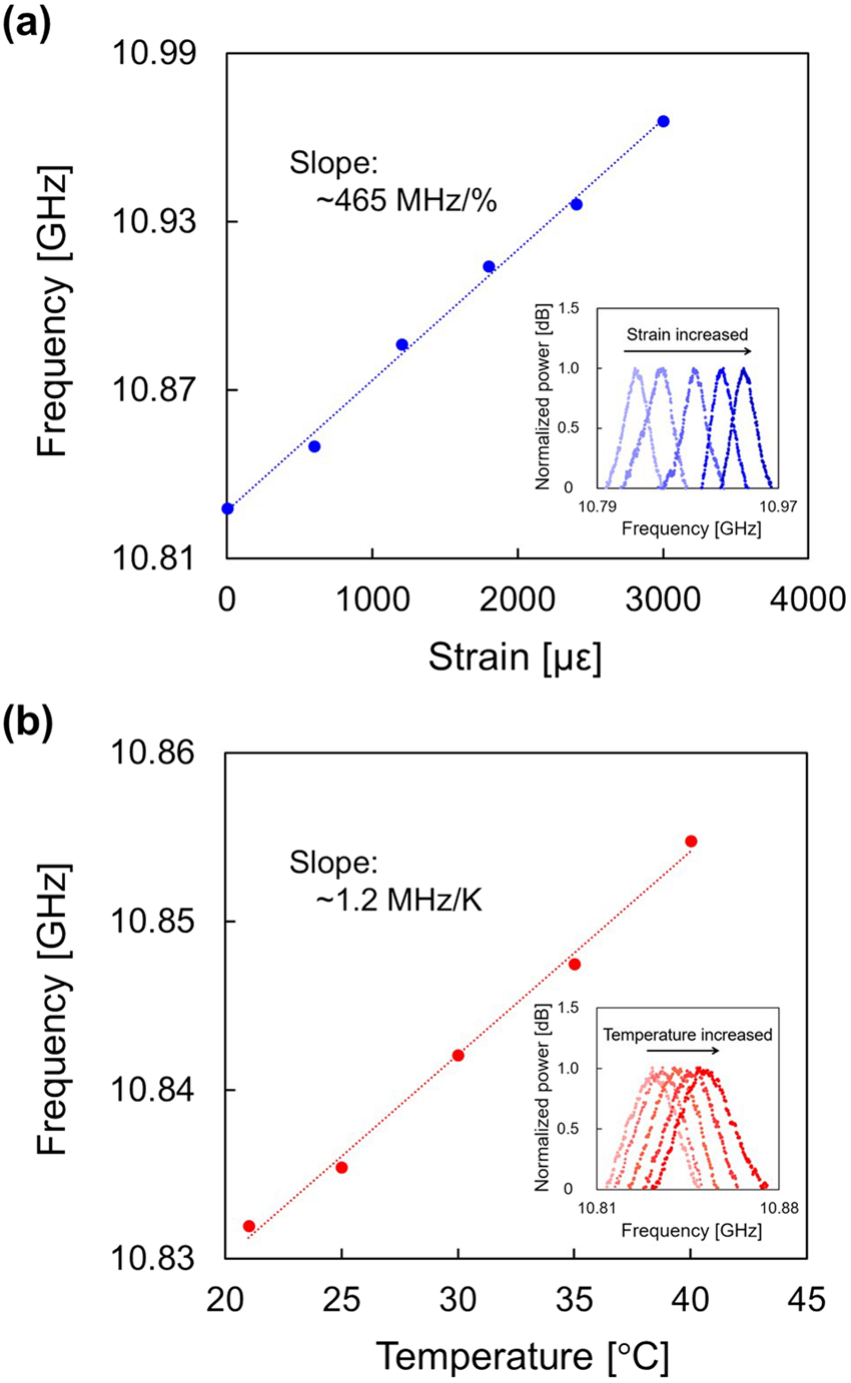
Measured dependencies of the BGS (insets) and the BFS on (**a**) strain and (**b**) temperature. Each BGS was normalized so that its maximal power became 1. The BFS was extracted from the BGS using Lorentzian fitting. The dotted lines are linear fits.

First, without modulating the optical frequency of the laser output (i.e., using the whole length of the FUT), the strain and temperature dependencies of the BGS and BFS in the low-bending-loss fiber were investigated. As shown in Fig. 2(a), with increasing strain, the BGS shifted to higher frequency (see the inset), and the BFS linearly depended on strain with a coefficient of 465 MHz/%. In the same manner (Fig. 2(b)), with increasing temperature, the BGS also shifted to higher frequency (see the inset), and the BFS dependence coefficient on temperature was 1.2 MHz/K. The strain and temperature dependence coefficients are 0.9 and 1.1 times the values in standard silica fibers reported in refs^37,38^; the small discrepancies are natural considering that the fiber cores are both composed of germanium-doped silica. Note that Brillouin bandwidth Δ*v*_B_ of this special fiber (non-strained, at room temperature) was approximately 40 MHz, which was used to calculate the nominal spatial resolution of the system using this fiber (see Eq. (2)).

**Figure 3:**
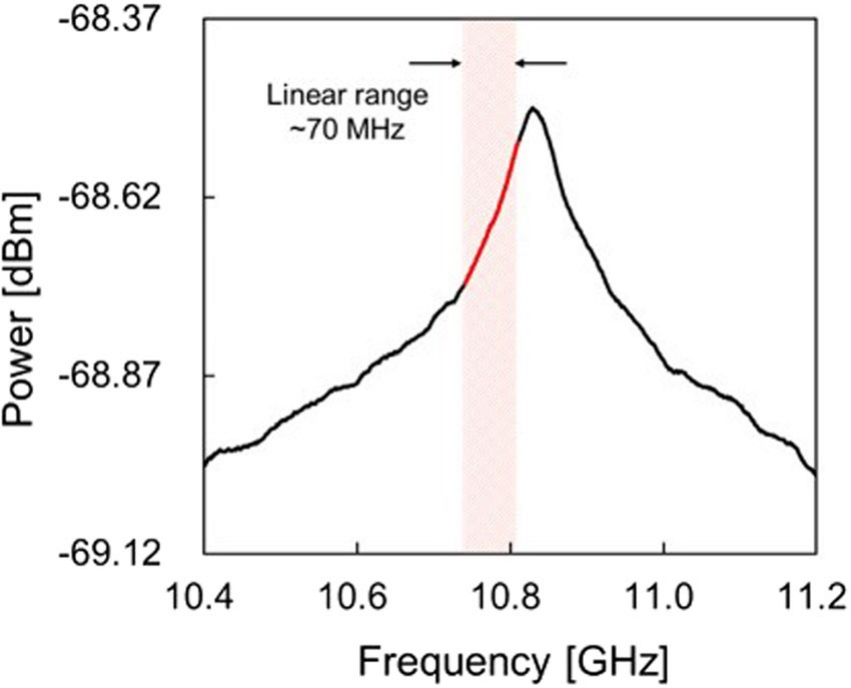
BGS measured when the spatial resolution was 144 mm and the measurement range of 14.7 m. The linear range on the lower-frequency side is indicated in red.

**Figure 4:**
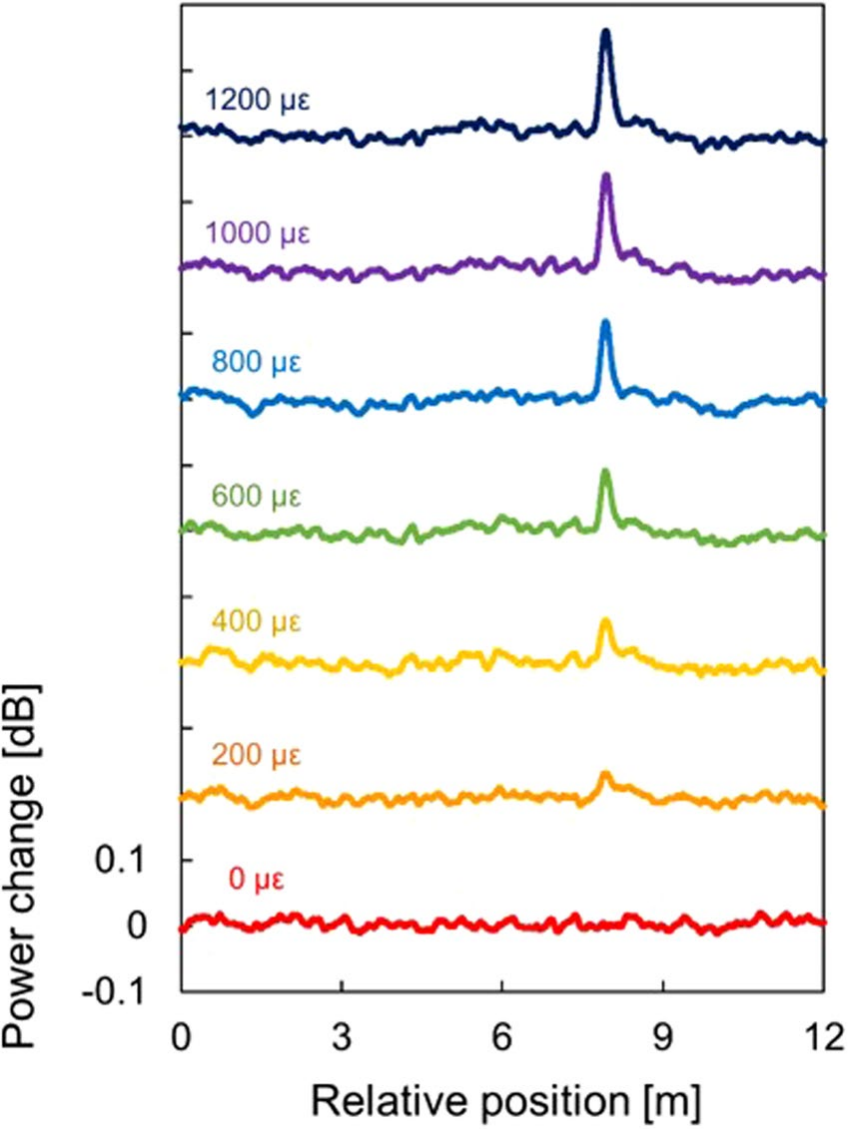
Measured strain dependence of the power-change distributions. Each distribution is shifted by 0.2 dB.

**Figure 5:**
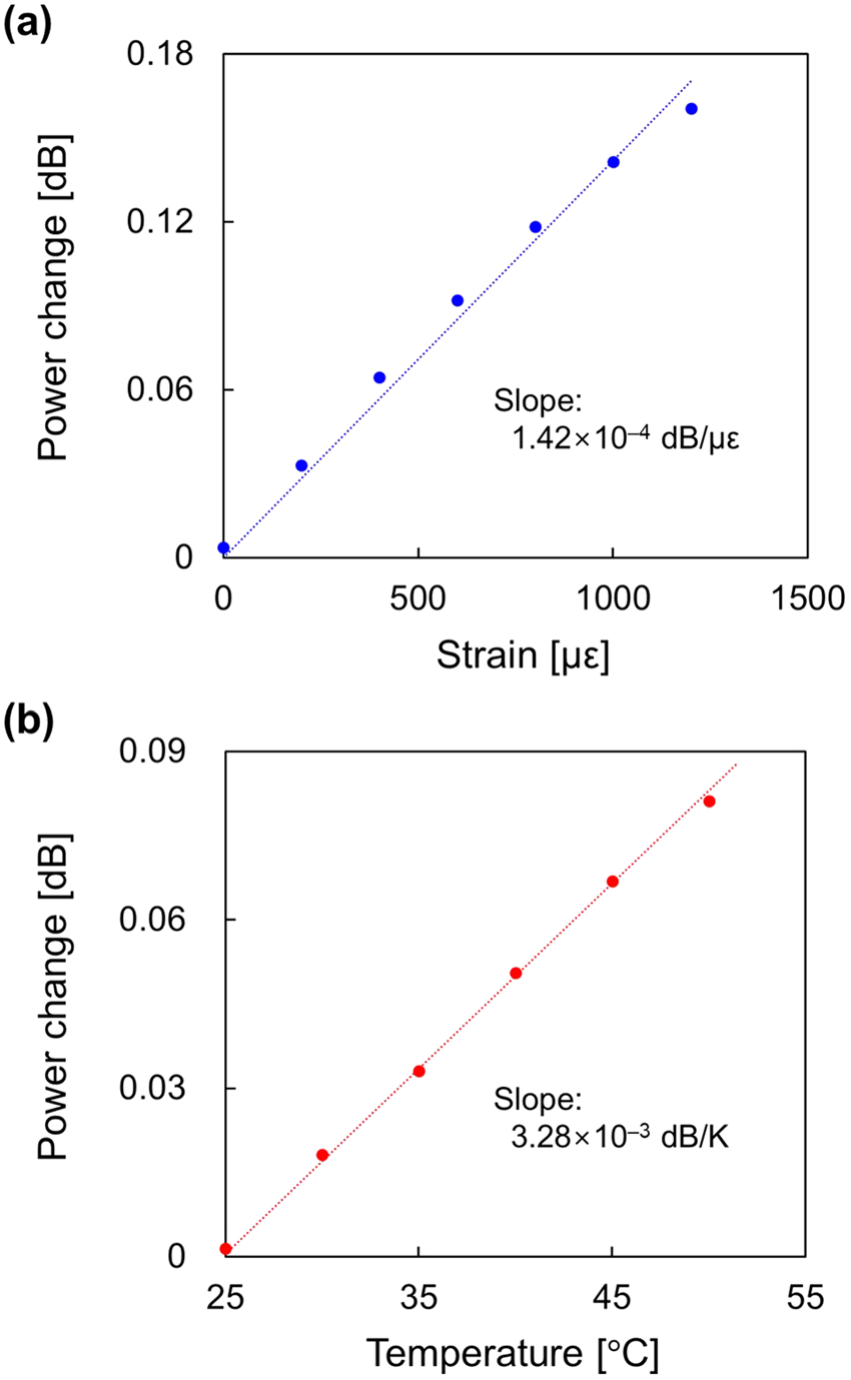
Measured power-change dependencies on (**a**) strain and (**b**) temperature. The dotted lines are linear fits.

Next, we performed a single-point measurement of strain and temperature to evaluate the sensing performance of SA-BOCDR using the low-bending-loss fiber. The measured BGS is shown in Fig. 3. By differentiating the BGS with respect to frequency, the optimal *v*_B0_ value was calculated to be 10.81 GHz, and the linear range (defined as the range where the change in the BGS slope is suppressed within 20% compared to its maximum)^30^ was calculated to be ~70 MHz. We then applied strains of up to 1200 με to the 0.15-m-long section (at the midpoint of which the correlation peak was located) and measured the dependencies of the power-change distributions (Fig. 4). Each distribution was displayed with an artificial shift of 0.2 dB for clear comparison. At the strained position, a clear peak was observed, and its peak amplitude increased with increasing strain. Refer to Methods for the reason why the power-change distributions do not form rectangular shapes at the strained position. Figure 5(a) shows the maximal power change plotted as a function of the strain. The dependence was almost linear with a coefficient of 1.42 × 10^−4^ dB/µε; the slight discrepancy from the linear trend seems to have been caused by the imperfectly straight BGS slope even within the linear range. The power-change dependence on temperature (<50 °C) was investigated under the same conditions, and as shown in Fig. 5(b), an almost linear behavior with a coefficient of 3.28 × 10^−3^ dB/K was obtained. These values were in moderate agreement with the theoretical values calculated using the BGS shape and the strain and temperature dependence coefficients of the BFS.

### Demonstration of bending-insensitive distributed measurement

**Figure 6:**
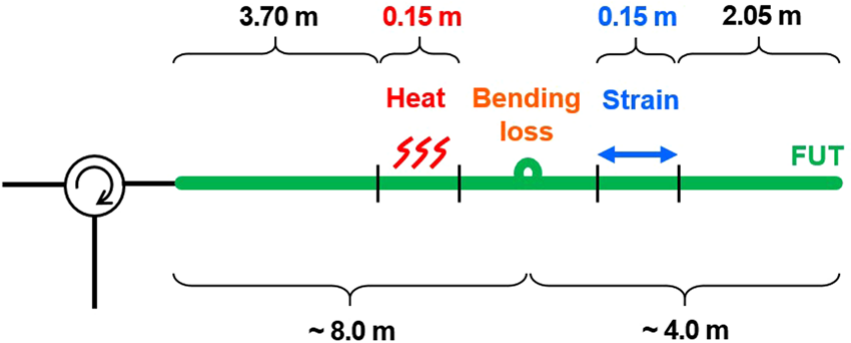
Structure of the FUT, where heat, strain, and bending loss were applied.

Finally, the loss-insensitive operation of SA-BOCDR was demonstrated using the FUTs depicted in Fig. 6. Two different types of 12.0-m-long silica SMFs (a standard SMF (FPC-SM20, Alnair Labs) and the aforementioned low-bending-loss SMF) were employed. Heat (50 °C) and strain (600 µε) were applied to 0.15-m-long sections, and bending losses were applied between the heated and strained sections. The bending loss was applied by winding the fibers around rods for one turn without strain. The radii of the rods were 8 and 10 mm, which theoretically induce bending losses of ~3.1 and ~0.8 dB for the standard SMF, respectively^40^. The bending losses induced to the special SMF were relatively low (<0.02 dB).

**Figure 7:**
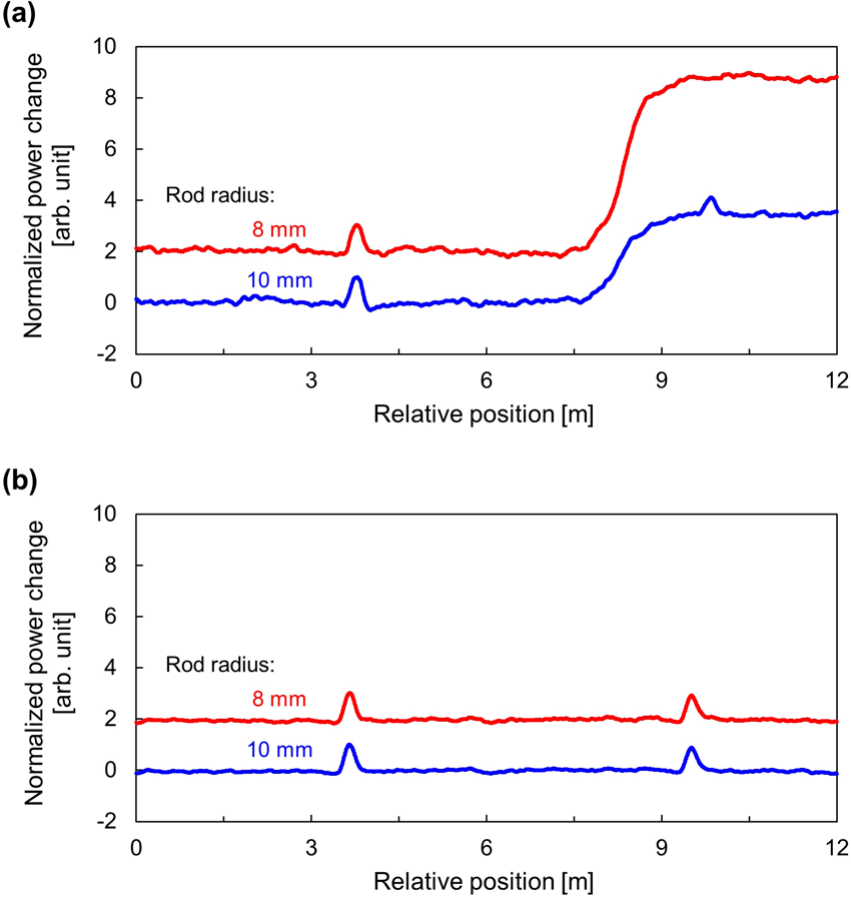
Normalized power-change distributions with rod radii of 8 and 10 mm when the FUT was (**a**) the standard silica SMF and (**b**) the low-bending-loss silica SMF. Each distribution is shifted by 2.

Figure 7(a) shows the power-change distributions measured along the standard SMF with 8- and 10-mm rod radii. The vertical axis was normalized so that the maximal power change at the heated section became 1 (when the rod radius was 10 mm). Note that the two distributions were displayed with an artificial shift of 2 for clear comparison. Regardless of the rod radii, the heated sections were correctly detected, and the rough locations of the applied bending losses were also detected (Refer to Methods for the reason why the power-change distributions do not form rectangular shapes at the heated/strained positions). However, when the rod radius was 10 mm, the location of the strained section was correctly detected, but the strain magnitude appeared to be much smaller than the actual value (which is approximately 0.6). This is because the BGS power reduced by bending loss lead to the reduction in the spectral slope, resulting in the reduced sensitivity to strain. When the rod radius was even smaller (8 mm), the loss was so high that the strained section was not detected at all. This is because the considerable loss almost completely diminished the BGS (i.e., the spectral slope became almost flat), and consequently, the strain-induced spectral shift posed only negligible influence on the final output. In contrast, Fig. 7(b) shows the normalized power-change distributions measured along the low-bending-loss SMF with 8- and 10-mm rod radii. The two distributions were similar to each other irrespective of the rod radii, and the heated and strained sections were correctly detected in both measurements. The applied bending losses did not affect the measured results, indicating that, by SA-BOCDR using this special fiber, a stable distributed measurement of strain and temperature can be performed with no influence of unintended local loss.

## Conclusion

In this work, we verified the loss-insensitive operation of SA-BOCDR using the special SMF. First, we measured the coefficients of the power-change dependencies on strain and temperature to be 1.42 × 10^−4^ dB/µε and 3.28 × 10^−3^ dB/K, respectively. Then, by comparing the distributed strain and temperature measurement results using the standard SMF and the special SMF, we proved the effectiveness of this loss-insensitively configured SA-BOCDR. To enhance the measurement stability further, the use of low-bending-loss polarization-maintaining fibers may be an option. We anticipate this configuration with high stability is of significant use in practically employing SA-BOCDR for structural health monitoring in the future.

## Methods

### Unique final output of SA-BOCDR

The final output of SA-BOCDR is a power-change distribution along an FUT, where the power change gives information of strain, temperature, and loss^30^. However, the power-change distribution obtained using SA-BOCDR is not in one-to-one correspondence to the BFS distribution (the correct BFS information can be derived, provided the SNR is sufficiently high)^34^. When strain or temperature change is uniformly applied to a section, the BFS distribution should form a rectangular shape, while the power-change distribution in SA-BOCDR basically exhibits a trapezoidal shape. In particular, when the length of the strained or heated section is equal to the nominal spatial resolution, it shows a triangular shape. Even when the strained or heated section is shorter than the nominal resolution, a trapezoidal-shaped shift in the power change can be observed, which is called the beyond-nominal-resolution effect^34^. This effect, which neither standard BOCDR systems nor SA-BOTDR/BOTDA systems^41–43^ can offer, is unique to SA-BOCDR and will be of great use in practical applications^30^. See ref.^34^ for the physical origin of this unique final output of SA-BOCDR.
